# Involvement of endoplasmic reticulum stress in trigeminal ganglion corneal neuron injury in dry eye disease

**DOI:** 10.3389/fnmol.2023.1083850

**Published:** 2023-03-24

**Authors:** Jinyu Zhang, Hongbin Lin, Fengxian Li, Kaili Wu, Shuangjian Yang, Shiyou Zhou

**Affiliations:** ^1^State Key Laboratory of Ophthalmology, Zhongshan Ophthalmic Center, Sun Yat-sen University, Guangdong Provincial Key Laboratory of Ophthalmology and Visual Science, Guangzhou, China; ^2^Department of Anesthesiology, Zhujiang Hospital of Southern Medical University, Guangzhou, China; ^3^Guangdong Institute for Vision and Eye Research, Guangzhou, China

**Keywords:** dry eye disease, environmental dry eye disease, corneal neuron RNA-sequencing, endoplasmic reticulum stress, trigeminal ganglion corneal neuron injury

## Abstract

Dry eye disease (DED) is a multifactorial disease with a high prevalence worldwide. Uncomfortable corneal sensations severely affect daily life in DED patients. Hence, corneal neuron injury is a vital pathogenesis in DED. Notably, endoplasmic reticulum stress (ERS) plays a role in peripheral neuron injury. However, the role of ERS in DED corneal neuron injury is still far from being clear. In this study, we established an environmental DED (eDED) model *in vivo* and a hyperosmotic DED model *in vitro*. Subsequently, trigeminal ganglion (TG) corneal neurons were retrograde labeled by WGA-Alexa Fluor 555, and fluorescence-activated cell sorting was used to collect targeted corneal neurons for RNA sequencing in mice. Our results revealed that there is TG corneal neuron injury but not neuron apoptosis in DED. ERS-related genes and proteins were upregulated in TG corneal neurons of the eDED mice. ERS inhibition alleviated TG corneal neuron’s ERS-related injury. Therefore, ERS-induced TG corneal neuron injury may be an important pathomechanism and provide a promising therapeutic approach to DED.

## 1. Introduction

Dry eye disease (DED) is a chronic disease of the ocular surface characterized by a loss of homeostasis of the tear film and accompanied by ocular symptoms, such as blurring vision, red eye, and neurosensory abnormalities (Craig et al., 2017). More than a billion people worldwide suffer from DED. Notably, environmental DED (eDED) is becoming more common, with an incidence of 35–48% (Stapleton et al., 2017). Severe DED leads to significantly decreased and even loss of vision, which seriously decreases the quality of life.

Cornea is the most densely innervated tissue in the body, with roughly 19,000–44,000 fibers distributed in the corneal subbasal nerve plexus (Muller et al., 1997). Indeed, corneal neurosensory abnormality is the most common symptom in DED which includes foreign body sensations, dryness, irritation, itching, and burning (Bron et al., 2017). Discomforting corneal sensations are mainly conducted by the corneal nerve from fibers inside the cornea to cell bodies in the trigeminal ganglion (TG), and are finally perceived in the higher brain center (He and Bazan, 2016). Neuronal injury leads to abnormal nerve conduction function. However, the role of TG corneal neuron injury and the potential mechanism of DED remain unclear.

The endoplasmic reticulum (ER) mainly controls cellular protein synthesis, folding, and structural maturation. The ER environment is sensitive to stresses that impair the folding of ER proteins (Belmont et al., 2010; Oakes and Papa, 2015). Strikingly, overwhelmed ER protein folding induces ER stress (ERS). Initially, slight ERS leads to cell survival, but severe ERS causes cell damage and apoptosis (Oakes and Papa, 2015; Wang et al., 2015). ERS exists in a pathological state in various tissue and cell types (McMillan et al., 1994; Lee et al., 2015). Notably, it plays an important role in neuron injury, which contributes to motor neuron damage in amyotrophic lateral sclerosis (Tadic et al., 2014). Previous studies have reported that ERS leads to corneal epithelium injury in dry eye conditions (Wang et al., 2016; Cho et al., 2019), suggesting DED carries an irreversible ERS in the corneal epithelium. However, it is unclear whether ERS is involved in TG corneal neuron injury in DED.

Here, we investigated the role of ERS in TG corneal neurons in DED. TG corneal neurons were collected by retrograde tracing for RNA sequence analysis from eDED and control mice. Our data demonstrates TG corneal neuron injury in DED via corneal neuron ERS, and our study provides an important mechanism of ERS in TG corneal neuron dysfunction and a potential therapeutic approach to DED.

## 2. Materials and methods

### 2.1. eDED model and treatments

Female C57BL/6J mice (5–6 weeks, 14–17 g) were purchased from Bestest Biotechnology Co., Ltd, Guangdong, China. All mice were housed for 12 h in light/dark cycle conditions and were given adequate food and water. The mice were quarantined and acclimatized for one week before experimentation. All procedures were conducted in accordance with national legislation and associated guidelines, approved by the Institutional Animal Care and Use Committee at Zhongshan Ophthalmic Center at Sun Yat-sen University (Ethics no. 2020-158).

An eDED mice model was built by a controlled dry system (CDS) according to our previous study (Chen et al., 2021). Briefly, mice in the dry eye group were reared in the CDS with a relative humidity of less than 20 ± 2%, with the air flow maintained at 3–3.5 m/s and temperature at 21 ± 1°C. The humidity in the control group was maintained at 55 ± 5%. For ERS inhibition assays, CDS and control (non-CDS treated) mice were intraperitoneally injected with 4-Phenylbutyric acid (4-PBA, MedChemExpress, HY-15654, USA) (20 mg/kg/day, 15 days). A second control group of CDS-treated mice was injected with the vehicle (PBS).

### 2.2. Primary culture of mice TG cells and treatments

Female C57BL/6J mice (5–6 weeks, 14–17 g) were anesthetized with 1% pentobarbital sodium and then sacrificed by cervical dislocation. TG tissue was collected and dissociated with 2.5 mg/ml collagenase I (Invitrogen, 17100-017, USA) at 37°C for 90 min followed by 0.17% Trypsin-EDTA (Gibco, 25200-056, USA) and 0.2% DNase I (Roche, 10104159001, Swit) incubation at 37°C for 15 min (Launay et al., 2016). Cells were filtered by 40 μm cell filtration, resuspended in Neurobasal A medium (Gibco, 1088802, USA) containing 5% fetal bovine serum (FBS, Gibco, 1027-106, USA), 2% B-27 supplement (Gibco, 17504044, USA) and 200 mM L-sodium glutamate (Gibco, 25030-149, USA) and plated on coverslips in 24 well plates.

After 2 days in culture, the TG cells were incubated (for the hyperosmotic DED model *in vitro*), with 0 and 109 mM NaCl (corresponding to osmolarities of 312, 450 mOsm, respectively) for 24 h. Osmolarity values were measured by an automatic cryoscopic osmometer (Loser, OM819, Germany). For ERS inhibition assays, 1 mM 4-PBA or 1 μM Bip inducer-X (BIX, MedChemExpress, HY-110188, USA) were pre-incubated for 6 h followed by hypertonic treatment in 450 mOsm. PBS was added to the vehicle group as a control.

### 2.3. Corneal fluorescein staining

Corneal fluorescein staining in anesthetized mice was performed by applying 0.5 μl of 0.25% fluorescein sodium into the inferior conjunctival sac of the eyes. Then, the cornea was examined by a slit lamp biomicroscope (66 Vision-Tech, YZ5T, China) with cobalt blue light. The corneal fluorescein sodium staining score was based on the area of corneal staining (Suwan-apichon et al., 2006). In brief, Grade 0, no punctate staining in the corneal area; Grade 1, corneal stained area ≤1/8; Grade 2, corneal stained area ≤1/4; Grade 3, corneal stained area ≤1/2; Grade 4, corneal stained area >1/2.

### 2.4. Mechanical intensity of the cornea

The mechanical intensity of the cornea was measured using von Frey Hairs (XiYao, NC127756, China) (Fakih et al., 2019), which reflects corneal sensitivity. Different forces of calibrated von Frey filaments (0.008, 0.02, and 0.04 g) were applied to the central cornea of the mice, as previously reported (Joubert et al., 2019). In brief, each cornea was tested five times with each filament, and the time interval of each test was more than 5 s. A positive response was recorded when the animal blinked ≥3 times when tested. All experiments were performed by the same investigator.

### 2.5. Hematoxylin and eosin (HE) staining

The right eyeball was isolated and fixed in FAS Eye Fixation Fluid (Servicebio, G1109-100ML, China) before paraffin embedding. The eyeballs were cut through cornea and optic nerve vertically, each section was 5 μm-thick. HE staining was employed to assess the integrity of corneal epithelium. The sections were imaged using Pannoramic Scan (3DHISTECH, Pannoramic MIDI, Hungary) and analyzed by Image J2 (v.1.53, National Institutes of Health, Bethesda, MD, USA).

### 2.6. Immunofluorescence staining and TUNEL assay

The mice were perfused with precooled 4% paraformaldehyde (PFA, Biosharp, BL539A, China) after being deeply anesthetized. TG tissue and eyeballs were removed and fixed with 4% PFA at room temperature for 24 h, then infiltrated with 20% sucrose. Next, TG tissues and eyeballs were embedded in an optimal cutting temperature compound (OCT, Tissue-Tek, Sakura Finetek, USA) followed with cryosections of 14 μm thickness. As for primarily cultured TG cells, TG cells on coverslips were fixed with 4% PFA at room temperature for 30 min, and then washed with PBS.

For immunofluorescence staining, each section and primary cultured TG cells were blocked in 10% goat serum (Boster, AR0009, China) at room temperature for 30 min. The primary and secondary antibodies used were as follows: rabbit anti-ATF3 (1:300, Abcam, ab244268, USA), mouse anti-NeuN (1:500, Merck Millipore, MAB377, Germany), rabbit anti-GRP78 (1:1,000, Abcam, ab21685, USA), rabbit anti-XBP-1s (1:1000, Cell Signaling Technology, 40435, USA), anti-rabbit IgG (H + L), F(ab’)2 Fragment (Alexa Fluor^®^ 488 Conjugate) (1:1,000, CST, 4412S, USA), and anti-mouse IgG (H + L), F(ab’)2 Fragment (Alexa Fluor^®^ 555 Conjugate) (1:1,000, CST, 4409S, USA). One Step TUNEL Apoptosis Assay Kit (Beyotime, C1086, China) was used to perform TUNEL assay. Next, sections were imaged using a confocal microscopy (Nikon, AX, Japan) and analyzed using NIS-Elements software (v.5.21.00, Laboratory Imaging s.r.o., Czechia). One or three different fields of view (the magnification power of each filed is 200 or 400) were selected from each section, and the number of positive cells in each field was counted. Then, the average number of positive cells per section was calculated (Wang et al., 2017). The same image settings were used for imaging the sections with anti-GRP78 and anti-XBP-1s. Fluorescence intensities of GRP78 and XBP-1s were analyzed with Image J2 (He et al., 2020).

### 2.7. Corneal nerve fiber staining

Corneas were collected from the right eye and fixed with methyl alcohol at room temperature for 1 h. Primary and secondary antibodies used were as follows: rabbit anti-TUBB3 (Sigma, T2200, USA), anti-rabbit IgG (H + L), F(ab’)2 Fragment (Alexa Fluor^®^ 488 Conjugate) (1:1000, CST, 4412S, USA). Whole mounts of corneal nerve fibers stained with TUBB3 (primarily expressed in neurons) were imaged using a fluorescent microscope (Nikon, Ds-Ri2, Japan). The corneal nerve fibers density was analyzed with Image J2 (He et al., 2020).

### 2.8. Quantitative RT-PCR (qPCR)

Real-time RT-qPCR was performed to measure the expression of GRP78, GRP94, XBP1, and CHOP mRNA of mouse TG tissue. GAPDH used as a housekeeping gene. Primer sequences are listed in Table 1. An RNAiso Plus kit (TaKaRa, 9109, Japan) was used to extract total RNA from TGs. A SYBR Green kit (Takara, RR820A, Japan) applied for qPCR using 2 μl cDNA.

**TABLE 1:** Genetic-specific primers for qPCR.

Gene	Forward primer	Reverse primer
ATF3	CGAGCGAAGACTGGAGCAAAATGA	AGGTGAGAGGCAGGGGACAAT
GRP78	ATCCCGTGGCATAAACC	CCAAGTGTAAGGGGACAAA
GRP94	AGGGAGAAGAGATGGATGC	TTTTACAGTGCAGGGGAGA
XBP-1	CTGCGGAGGAAACTGAAA	CTTCCAAATCCACCACTTG
CHOP	GGAGCTGGAAGCCTGGT	GGATGTGCGTGTGACCTCT
GAPDH	GGCAAATTCAACGGCACAGTCAAG	TCGCTCCTGGAAGATGGTGATGG

### 2.9. TG corneal neuron cell body retrograde labeling

Mice were anesthetized and placed under a stereoscopic dissection microscope. Wheatgerm agglutinin-Alexa Fluor 555 (WGA-AF555, Invitrogen, W32464, USA), a retrograde neural tracer, was used to label TG corneal neurons. Approximately 0.5 μl WGA-AF555 of 5 mg/mL in PBS was injected into the corneal stroma via a glass micropipette (Li et al., 2019). Five days after WGA-AF555 injection, eDED group mice were held under CDS conditions for 14 days.

### 2.10. Fluorescence-activated cell sorting assay (FACS)

According to section “2.2. Primary culture of mice TG cells and treatments,” TG tissue of WGA-AF555 retrograde tested mice or PBS tested mice was dissociated to single cells. Cells were filtered by 40 μm cell filtration, resuspended in PBS containing 0.2% FBS. Cell suspension was then sorted using a flow cell sorter (BD, FACSAria Fusion, USA) set up with a 130 μm nozzle (Ivanov et al., 2008). WGA-AF555 positive neurons were defined by electronic gating in FACSDiva software (BD, v.8.01, USA) using forward and side-angle light scatter, and AF561 fluorescence. Fluorescence minus control was used to set voltage and gate for WGA-AF555 positive neurons. Data was analyzed using Flowjo (Treestar, v.10.8.1, USA). The separated WGA-AF555 labeled neurons were then collected for subsequent experiments.

### 2.11. Western blot

Trigeminal ganglion from each mouse was homogenized with a tissue grinder (Servicebio, KZ-III-FP, China), and total protein was extracted using RIPA buffer (Beyotime, P0013B, China) according to the manufacturer protocol, containing 1% phenylmethylsulfonyl fluoride (Beyotime, ST506, China). The protein concentration was measured by BCA protein assay kit (Beyotime, P0010S, China). Equal amounts of protein were then subjected to electrophoresis on 12% SurePAGE, Bis-Tris gels (Genscript, M00669, USA) followed by electrophoresis transfer to PVDF membranes (Millipore, IPVH00010, USA). Membrane blocking was performed with 5% skim milk (BD, 232100, USA) at room temperature for 2 h. Primary antibodies were used rabbit anti-GRP78 (1:1,000, Abcam, ab21685, USA), and anti-GAPDH (1:20,000, Proteintech, 10494-1-AP, China). Secondary antibody used was goat anti-rabbit IgG (1:10,000, Cell Signaling Technology, 7074, USA). The Omni-ECL femto light chemiluminescence kit (EpiZyme, SQ201, China) was used to visualize the bands. Immunoreactivity was detected using automatic chemiluminescence (Tanon, 5200, China). Image J2 was used to semi-quantify the blot images.

### 2.12. Smart-seq2 RNA sequencing

#### 2.12.1. cDNA and sequencing library preparation

Total RNA of TG corneal neurons was extracted using trizol reagent (Invitrogen, 15596018, USA) according to the manufacturer protocol. RNA quality was assessed on an Agilent 2100 Bioanalyzer (Agilent Technologies, USA) and checked using RNase free agarose gel electrophoresis. Following total RNA extraction, eukaryotic mRNA was enriched by Oligo (dT) beads. The cDNA libraries were sequenced on the Illumina sequencing platform by Genedenovo Biotechnology Co., Ltd (Guangzhou, China). Reads were further filtered by fastp (v.0.18.0) before analysis (Chen et al., 2018).

#### 2.12.2. Differential expression calling and KEGG enrichment analysis

RNA differential expression analysis of TG corneal neurons between the control and eDED groups was performed by DESeq2 (Love et al., 2014) software and by edgeR between two samples (Robinson et al., 2010). Those transcripts with the parameters *P* < 0.05 and absolute fold change ≥ 1.2 were considered differentially expressed. The Kyoto Encyclopedia of Genes and Genomes (KEGG) is the major public pathway-related database (Love et al., 2014). Pathway enrichment analysis identified significantly enriched metabolic pathways or signal transduction pathways in differentially expressed genes compared with the whole genome background.

### 2.13. Statistical analysis

Data are shown as mean ± SEM and analyzed using Prism8 software (GraphPad8, San Diego, CA, USA). Statistical significance was determined using the Student t-test or one-way ANOVA followed by a Tukey Kramer *post hoc* test or repeated ANOVA followed by a least significance difference (LSD) *post hoc* test, as appropriate. The difference between the groups was considered statistically significant at *P* < 0.05.

## 3. Results

### 3.1. eDED induced corneal epithelium injury

**FIGURE 1:**
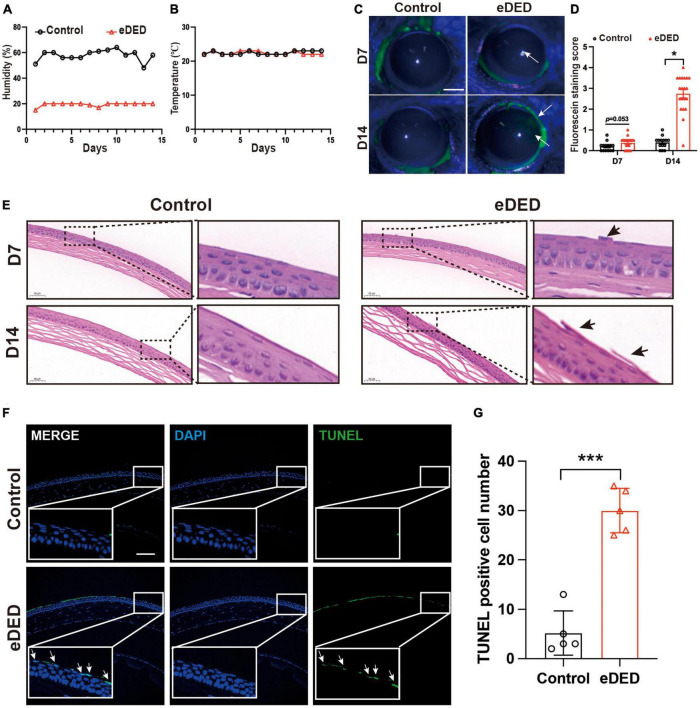
Corneal epithelium injury in environmental dry eye disease mice. **(A)** Decreased mean relative humidity recordings in the CDS (20 ± 2%) compared to the vivarium (55 ± 5%) throughout the experiment. **(B)** Mean temperature (21 ± 1°C) recordings in the CDS and vivarium throughout the experiment. **(C)** Increased corneal epithelium defective area is indicated by corneal fluorescein sodium staining (green, arrows) was shown in eDED mice on day 7 and 14; scale bar: 1 mm. **(D)** The score of corneal fluorescein sodium staining in control (*n* = 15) and eDED mice (*n* = 17), groups on day 7 and 14. **(E)** Increased desquamation of apical corneal epithelial cells (arrows) shown by corneal HE staining in eDED mice on day 7 and 14; scale bar: 50 μm. **(F)** Representative images of corneal epithelium showing an increase in apoptotic cells (arrows) in eDED mice via TUNEL assay on day 14. Co-localization of TUNEL (green, arrows) and DAPI (blue); scale bar: 100 μm. **(G)** Quantification of TUNEL positive cells in corneal epithelium in the control and eDED group on day 14 (*n* = 5 in each group); Y-axis represents TUNEL positive cell number. Each dot represents mean TUNEL positive cells of three different fields of view (the magnification power of each field is 200) per corneal slice. Data are represented as mean ± SEM; *P < 0.05, ***P < 0.001; CDS, controlled dry system; eDED, environmental dry eye disease.

To establish an eDED mouse model, the housing room humidity and temperature in the CDS were maintained at 20 ± 2% and 21 ± 1°C, respectively (Figures 1A, B). Compared to the control group, the weight of eDED mice significantly decreased on day 14, and there was a decrease of tear secretion in eDED mice after 14 days housed in the CDS (*P* < 0.05) (Supplementary Figures 1A, B). Only corneal epithelium defective areas could be stained by fluorescein sodium, staining area was measured according to the scoring criteria (Suwan-apichon et al., 2006). Scores staining were found to be higher in the eDED group compared to the control, only on day 14 (Day 14: Control: 0.4 ± 0.3 point; eDED: 2.7 ± 0.9 point; *P* < 0.05) (Figures 1C, D). Desquamation of apical corneal epithelium cells was observed on day 7 and 14 in the eDED group (Figure 1E). eDED also induced significant corneal epithelium cell apoptosis on day 14, as detected by TUNEL assay (*P* < 0.001) (Figures 1F, G), compared to the control group which showed almost negative TUNEL fluorescence. Altogether, these results indicated that corneal epithelial cells were injured in the eDED group.

### 3.2. TG corneal neuron injury in DED

**FIGURE 2:**
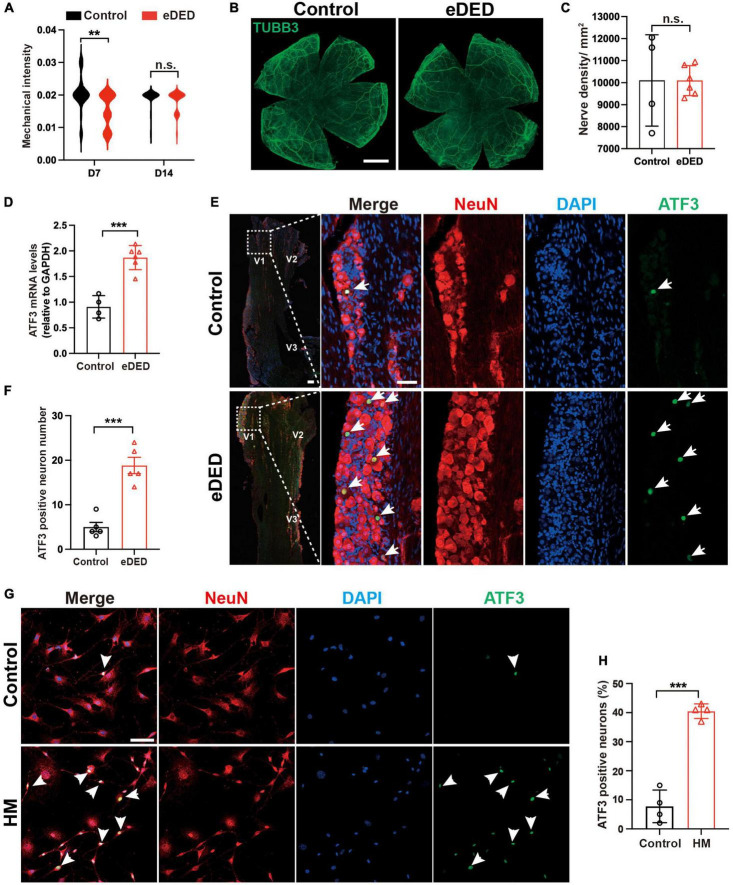
Trigeminal ganglion (TG) corneal neuron injury in dry eye disease. **(A)** Violin graph showing corneal sensitivity via mechanical intensity in the control (*n* = 22) and eDED (*n* = 30), groups on day 7 and 14. **(B)** TUBB3 is a marker of neural cell body and fibers membrane. No significant change is seen in cornea nerve fibers (TUBB3, green) in the whole mount cornea of the control and eDED mice on day 14; scale bar: 1 mm. **(C)** Quantification of cornea nerve fiber density (count per mm^2^) in control (*n* = 4) and eDED (*n* = 6) groups. **(D)** Quantification of TG ATF3 gene expression (relative to GAPDH mRNA) in control (*n* = 4) and eDED (*n* = 6), groups on day 14. **(E)** Increased TG neuron injury in the V1 part indicated by immunofluorescence staining in eDED mice on day 14. Co-localization of neuron injury marker ATF3 (expressed in nucleus, green, arrows), neuron marker NeuN (red) and DAPI (blue); scale bar, 50 μm. **(F)** Quantification of ATF3-positive neurons in the control and eDED groups on day 14 (*n* = 5 in each group). Y-axis represents the number of ATF3-positive neurons. Each dot represents the number of ATF3-positive neurons of one field of view (the magnification power of each field is 200) per TG slice. **(G)** Increased *in vitro* TG neurons injury indicated by immunofluorescence staining in HM group. Co-localization of neuron injury marker ATF3 (arrowheads), neuron marker NeuN and DAPI; scale bar, 50 μm. **(H)** The proportion of ATF3-positive neurons of TG cell slices in the control and HM groups (*n* = 4 in each group). Y-axis represents the proportion of ATF3-positive neurons. Each dot represents the mean ATF3-positive neurons proportion of three different fields of view (the magnification power of each field is 400) per cell culture dish. Data are represented as mean ± SEM; **P < 0.01, ***P < 0.001; n.s., not significant. DED, dry eye disease; eDED, environmental dry eye disease; V1, ophthalmic branch; V2, maxillary branch; V3, mandible branch; HM, hyperosmotic medium; TG, trigeminal ganglion.

One of the most important functions of the cornea is the perception of mechanical sensation. We found low corneal mechanical intensity on day 7 in the eDED group (Control: 0.0202 ± 0.0054 g; eDED: 0.0154 ± 0.0051 g; *P* < 0.01) (Figure 2A), which indicated increased corneal sensitivity. Compared to the control group, there are not significant difference in the mechanical intensity in eDED group on day 14 (Figure 2A). In general, corneal sensitivity is highly related to corneal nerve density. Corneal whole mount stained with TUBB3 (a marker of neural cell body and fibers membrane) was used to evaluated the corneal nerve fiber density. However, there was no significant difference in the corneal nerve fiber density between the control and eDED groups on day 14 (Figures 2B, C). Most corneal nerve fibers came from corneal sensation neuron, and the cell body located at TG (He and Bazan, 2016). Changes in mechanical sensation can be caused by neuronal cell body injury. ATF3 is a marker for injured neurons that is normally expressed in the nucleus. Therefore, ATF3 was used to evaluate the TG neuron injury. We found that the mRNA expression of ATF3 was highly upregulated in the eDED group than in the TG control group (*P* < 0.001) (Figure 2D). Likewise, the number of ATF3-positive neurons in the V1 part of TG increased (eDED vs. control, *P* < 0.001) (Figures 2E, F). However, there was no TG neuronal apoptosis in the eDED mice (Supplementary Figure 2A). The number of ATF3-positive TG cells was also increased when cultured in the hyperosmotic medium comparing to the control group *in vitro* (*P* < 0.001) (Figures 2G, H). These results demonstrated that TG corneal neurons were injured in the DED.

### 3.3. ERS in the TG corneal neurons in DED

**FIGURE 3:**
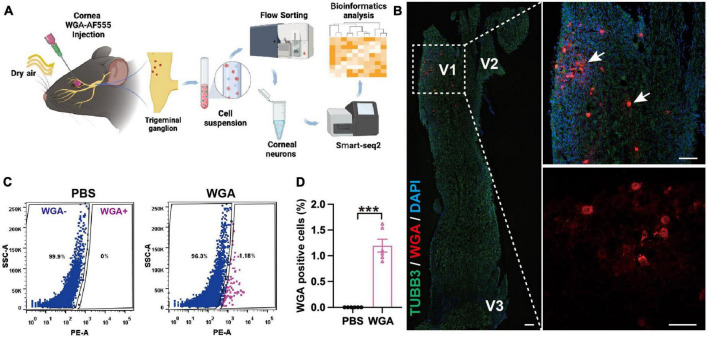
Trigeminal ganglion (TG) corneal neurons in environmental dry eye disease mice sorted for RNA sequencing. **(A)** Procedure flowchart showing, that after corneal WGA-AF555 injection, the mice were given CDS treatment and then TGs were FACS sorted, followed by corneal neurons RNA sequencing application. **(B)** Representative images of retrograde WGA-AF555 (red, arrows) corneal neuron cell bodies in TG on day 3. Scale bars: 100 μm. **(C)** Representative FACS images of WGA-AF555-positive corneal neurons in TG on day 3. Blue: WGA-AF555 negative cells, pink: WGA-AF555-positive cells. **(D)** The proportion of WGA-AF555-positive cells from a total TG population in the corneal WGA-AF555 injection (*n* = 6) and PBS injection (*n* = 6) groups. Data are represented as mean ± SEM; ***P < 0.001; WGA-AF555: wheatgerm agglutinin-Alexa Fluor 555, CDS, controlled dry system; eDED, environmental dry eye disease; FACS, fluorescence-activated cell sorting; TG, trigeminal ganglion; V1, ophthalmic branch; V2, maxillary branch; V3, mandible branch.

To determine the mechanism of TG corneal neuron injury, we collected TG corneal neurons from the eDED and control mice, and then applied RNA sequencing. WGA-AF555 is a retrograde fluorescent neural tracer. First, we injected the WGA-AF555 into the corneal matrix. Next, eDED mice were given CDS treatment. Then, we used FACS to separate the retrograde labeled WGA-AF555 neurons in the TG and collect them for smart-seq2 analysis for RNA sequencing (Figure 3A). The WGA-AF555-positive cells of the corneal neurons were mainly found to be clustered in the ophthalmic branch (V1 part) of TG (Figure 3B). Compared to the PBS injection group, the percentage of WGA-AF555-positive neurons separated by FACS was 1.2 ± 0.3% (*P* < 0.001) out of total TGs neurons in a normal mouse (Figures 3C, D) (total number of cells in TGs of a normal mouse is 4.5 × 10^4^ ± 3.8 × 10^3^) (Supplementary Table 2).

**FIGURE 4:**
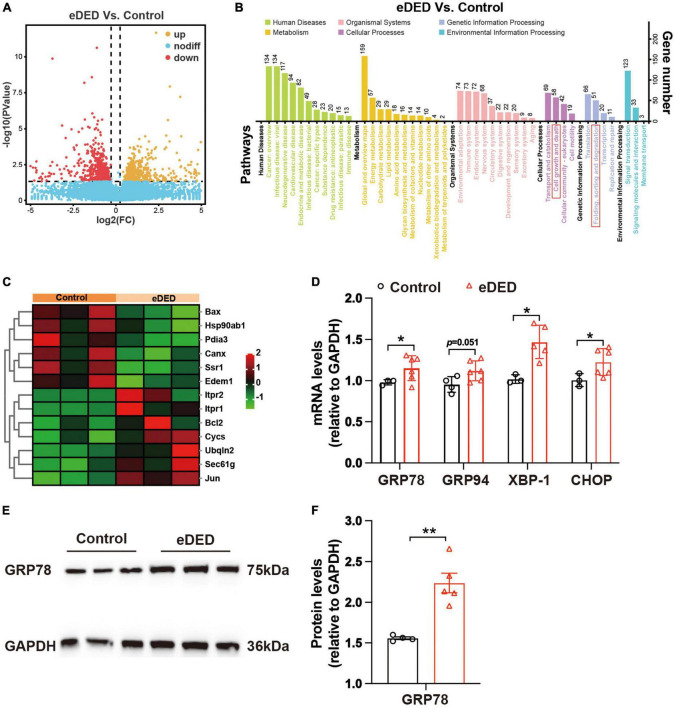
Trigeminal ganglion (TG) corneal neuron RNA sequencing analysis. **(A)** Volcano plot showing the different genes between the control and eDED groups on day 14 (*n* = 3 in each group). Yellow: upregulated genes, red: downregulated genes, blue: unchanged genes. **(B)** KEGG enrichment analysis in the control and eDED groups on day 14 (*n* = 3 in each group). The X-axis represents the pathways, and the Y-axis represents the gene number. The cell growth and death pathways as well as folding, sorting, and degradation pathways are marked with red rectangles. **(C)** Heat map of differentially expressed genes of ERS and apoptosis on day 14. Red: upregulation; green: downregulation. RNA sequencing analysis with the parameters of *P* < 0.05 and absolute fold change ≥ 1.2 were considered differentially expressed genes/transcripts. **(D)** Quantification of ERS and apoptosis-related genes (GRP78, GRP94, XBP-1, and CHOP) expression levels in TG on day 14. The measured RNA expression levels were normalized to GAPDH. **(E)** Increased ERS-related protein GRP78 expression indicated by western blot in eDED TG on day 14. **(F)** The integrated density value of GRP78 (relative to GAPDH protein) in the control (*n* = 4) and eDED (*n* = 5), groups on day 14. Data are represented as mean ± SEM; *P < 0.05, **P < 0.01; eDED, environmental dry eye disease; ERS, endoplasmic reticulum stress; TG, trigeminal ganglion.

Compared to the control group, there were 1,200 genes of different levels in TG corneal neurons in the eDED group, of which 600 were upregulated and 600 downregulated genes (Figure 4A). According to the KEGG pathways reactome, 1,200 different genes were separated into 43 KEGG pathways. Then, we focused on cell growth and death pathways (which includes 58 genes) as well as in folding, sorting, and degradation pathways (which includes 51 genes) (Figure 4B and Supplementary Table 1). Both categories contain ERS- and cell apoptosis-related genes. The ERS- and apoptosis-related gene expressions were significantly changed in TG corneal neurons in the eDED group comparing to the control group, including upregulated genes (Bax, Canx, Ssr1, Hsp90ab1, Pdia3) and downregulated genes (Itpr2, Ubqln2, Sec61g, Cycs, Edem1, Jun, Bcl2) (Figure 4C). qPCR analysis verified that the mRNA expression of ERS- and apoptosis-related genes like GRP78, GRP94, XBP1, and CHOP in TG was significantly elevated in the eDED compared to the control group (*P* < 0.05) (Figure 4D). Furthermore, the eDED ERS-related protein (GRP78) level in TG was increased when compared to the control group (*P* < 0.01) (Figures 4E, F). Collectively, our results suggested that ERS may relate to TG corneal neuron injury in eDED.

### 3.4. ERS is crucial to DED

**FIGURE 5:**
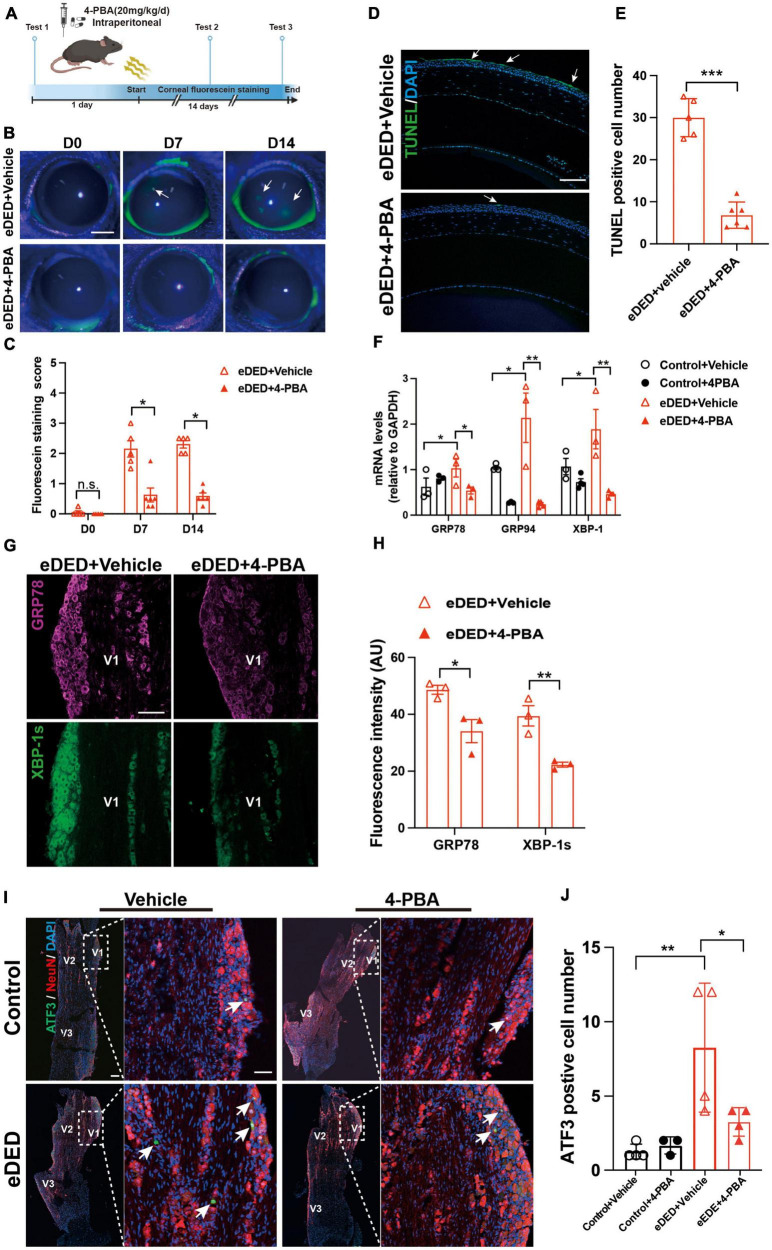
Decreased TG corneal neuron injury after inhibiting ERS in environmental dry eye disease. **(A)** Schematic diagram of the 4-PBA treatment procedure *in vivo*. Mice received 4-PBA (20 mg/kg/day) treatment once a day via intraperitoneal daily before and during CDS; corneal fluorescein testing was performed at three time points (day 0, 7, and 14). **(B)** Decreased corneal epithelium defective area shown by corneal fluorescein sodium staining (green, arrows) in eDED with 4-PBA treatment mice on day 0, 7, and 14; scale bar: 1 mm. **(C)** The score of corneal fluorescein sodium staining in eDED with (*n* = 5) and without 4-PBA treatment (*n* = 6), groups on day 0, 7, and 14. **(D)** Representative images of corneal epithelium shown decreased apoptotic cells (green, arrows) in eDED with 4-PBA treatment mice via TUNEL assay on day 14; scale bar: 100 μm. **(E)** Quantification of TUNEL positive cell number of corneal epithelium in eDED with (*n* = 5) and without 4-PBA treatment (*n* = 6), groups on day 14. Y-axis represents the number of TUNEL positive cells. Each dot represents mean TUNEL positive cells of three different fields of view (the magnification power of each field is 200) per corneal slice. **(F)** Quantification of ERS-related genes (GRP78, GRP74, and XBP-1) expression levels of TG in control with vehicle treatment group, control with 4-PBA treatment group, eDED with vehicle treatment and eDED with 4-PBA treatment group on day 14 (*n* = 3–5 in each group). The measured RNA expression levels were normalized to GAPDH. **(G)** Increased expression of ERS-related protein GRP78 (purple), and XBP-1s (green) in V1 part TG in eDED mice on day 14; scale bar, 100 μm. **(H)** The fluorescent intensity of GRP78 and XBP-1s positive cells in eDED with vehicle treatment, and eDED with 4-PBA treatment groups on day 14 (*n* = 3 in each group). **(I)** Decreased neuron injury of the V1 part TG indicated by immunofluorescence staining in eDED with 4-PBA treated mice on day 14. Co-localization of neuron injury marker ATF3 (green, arrows), neuron marker NeuN (red) and nucleus marker DAPI (blue); scale bar: 100 μm. **(J)** Quantification of ATF3-positive neurons in control with vehicle and 4-PBA treatment group, eDED with vehicle and 4-PBA treatment group on day 14 (*n* = 3–4 in each group). Y-axis represents the number of ATF3-positive neurons. Each dot represents mean ATF3-positive neurons of one field of view (the magnification power of each field is 200) per TG slice. Data are represented as mean ± SEM; *P < 0.05, **P < 0.01, ***P < 0.01, n.s., not significant; CDS, controlled dry system; eDED, environmental dry eye disease; ERS, endoplasmic reticulum stress; TG, trigeminal ganglion; V1, ophthalmic branch; V2, maxillary branch; V3, mandible branch.

**FIGURE 6:**
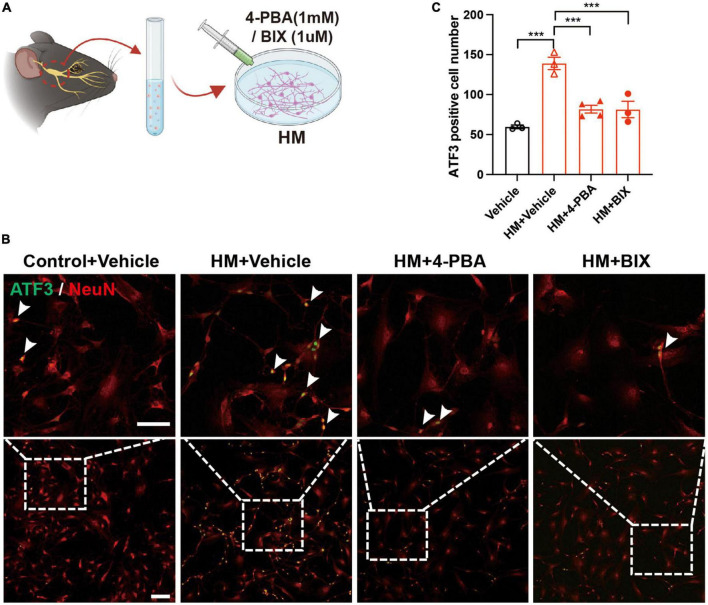
Decreased TG neuron injury of after inhibiting ERS in hyperosmotic DED. **(A)** Schematic diagram of the 4-PBA and BIX treatment procedure *in vitro*. Primary TG neurons were treated with 4-PBA (1 mM) or BIX (1 μM) in an HM culture. **(B)** Decreased neuron injury of TG indicated by immunofluorescence staining in HM with ERS inhibitor (4-PBA/BIX) treatment groups. Co-localization of neuron injury marker ATF3 (green, arrowheads) and neuron marker NeuN (red, arrowheads). Scale bars, 100 μm; **(C)** Quantification of ATF3-positive neurons in control with vehicle treatment group, HM with vehicle treatment group, HM with 4-PBA treatment group, and HM with BIX treatment group (*n* = 3–4 in each group). Y-axis represents the number of ATF3-positive neurons. Each dot represents mean ATF3-positive neurons of three different fields of view (the magnification power of each field is 200) per cell culture dish. Data are represented as mean ± SEM; ***P < 0.001; DED, dry eye disease; ERS, endoplasmic reticulum stress; HM, hyperosmotic medium; TG, trigeminal ganglion.

To evaluate the role of ERS in TG corneal neurons in DED, the eDED mice received 4-PBA (an ERS inhibitor) via intraperitoneal injection once a day for 15 days (Figure 5A). Compared with the vehicle treatment, the fluorescein staining scores were significantly lower on day 7 and 14 in 4-PBA treatment for the eDED mice (*P* < 0.05) (Figures 5B, C). Furthermore, 4-PBA treatment alleviated corneal epithelium cell apoptosis (Figures 5D, E). The mRNA expression of ERS associated genes (GRP78, GRP94, and XBP-1) in the 4-PBA group was significantly decreased (eDED + 4-PBA vs. eDED + vehicle, GRP78: *P* < 0.05, GRP94: *P* < 0.01, XBP-1: *P* < 0.01) (Figure 5F), as well as the expression level of ERS related proteins (GRP78 and XBP-1) which found to be reduced in the eDED + 4-PBA group, compared to the eDED-vehicle treated control treatment group (GRP78: *P* < 0.05 and XBP-1: *P* < 0.01) (Figures 5G, H). However, there was no TG neuronal apoptosis in the eDED mice with and without 4-PBA treatment (Supplementary Figure 2B). Furthermore, the number of ATF3-positive neurons was significantly reduced following 4-PBA treatment (eDED + 4-PBA vs. eDED + vehicle, *P* < 0.05) (Figures 5I, J). As expected, the application of ERS inhibitors (4-PBA and BIX) rescued the neuron injury of TG in hyperosmotic medium *in vitro* (all *P* < 0.001) (Figures 6A–C). Overall, these data suggested a dominant role of ERS in DED.

## 4. Discussion

In this study, ERS-induced TG corneal neuron injury was demonstrated in eDED mice, which may be the first sign of neuronal injury in DED. ERS was identified by RNA sequencing in eDED and then verified by qPCR in corneal neurons, which are located in the ophthalmic branch (V1 part) of TG. Noteworthy, ERS-related genes and proteins expression, as well as neuronal injury were significantly down-regulated under ERS-inhibition treatment. Therefore, this study revealed that ERS plays a crucial role in DED.

Environmental desiccation is an important risk factor of DED, which involves tear hyperosmolarity (Chen et al., 2013). Notably, eDED contributed nearly 50% of DED, which affected people’s daily life in varying degrees (Stapleton et al., 2017). Female are more susceptible to showing DED signs than male (Sullivan et al., 2017). Therefore, we used female mice to study the mechanism of DED. In this study, an *in vivo* mouse model was performed to imitate environmental desiccation, and an *in vitro* cell culture model was used to emulate tear hypertonic DED. Presently, dry eye animal models could be induced by lacrimal gland excision, scopolamine intracutaneous injection, genetical alteration and dry environment (Chang et al., 2021). Tear hyperosmolarity is common in all kinds of DED including eDED (Chen et al., 2013). The hyperosmotic cell culture model was frequently used to imitate DED *in vitro* (Li et al., 2004, 2006; Guzman-Aranguez et al., 2017). Therefore, a hyperosmotic cell culture *in vitro* system was chosen for supporting the dry eye animal model for better exploring the pathological mechanism of TG corneal neuron injury in DED.

Mechanical sensitivity was increased in the eDED mice in our study, in accordance to mechanical sensitivity increased in DED patients and DED models in previous studies (Spierer et al., 2016; Fakih et al., 2019), revealing corneal neuron dysfunction in eDED. Generally, corneal sensitivity is closely related to corneal nerve density (He et al., 2017). However, our results showed no change in corneal nerve fiber density in eDED. Likewise, clinical DED patients also showed no difference in corneal nerve fiber density compared to the healthy population (Shetty et al., 2016; Khamar et al., 2019). Notably, neuronal cell body damage also contributes to neurological dysfunction (Murthy et al., 2018). Sensitive corneal nerve fibers come from corneal sensation neuron, and the neural cell body is in TG (Muller et al., 1997). We demonstrated TG corneal neuron injury in eDED mice and *in vitro* hyperosmotic cell culture model, indicating that TG corneal neuron injury may lead to corneal sensation dysfunction in DED mice.

Notably, the corneal nerve not only enables sense nerve conduction but also plays an important role in the maintenance of a healthy ocular surface state, such as supporting healthy tear secretion, protective reflexes, and corneal epithelial and stromal cells by its secreted neurotrophic factors (Medeiros and Santhiago, 2020; Mittal et al., 2021). Also, cutting the ophthalmic branch of TG increases apoptosis and reduces the proliferation of epithelial cells (Ferrari et al., 2011). In this current study, the results showed that the corneal epithelium had defects in eDED, which is consistent with other studies (Barabino et al., 2005; Ortiz et al., 2020). When the corneal epithelium is injured or corneal ulcer exists in diabetic corneal neuropathy, increased corneal nerve sensitivity combined with corneal pain appears (Mansoor et al., 2020; Ren et al., 2020), suggesting that the abnormal corneal sensation can be attributed to corneal injury. During thermal or chemical stress, the corneal epithelium will secret adenosine triphosphate out of cells, which affects the signal conduction function of the corneal nerve (Veiga Moreira et al., 2007; Lapajne et al., 2020). Furthermore, neurotrophic factors, such as nerve growth factor and glial cell-derived neurotrophic factor released from the corneal epithelium can promote corneal nerve regeneration, suggesting their protective function of the corneal epithelium for the corneal nerve (Di et al., 2017). Therefore, the crosstalk between the corneal epithelium and TG corneal neurons may play an important role in the pathogenesis of DED.

RNA sequencing is an effective tool for the transcriptome-wide analysis of differential gene expression in cell or tissue samples (Stark et al., 2019). Previous studies on DED corneal neuron have focused on the whole TG (Fakih et al., 2019, 2021). However, TG contains a vast number of sensory neurons, including those from eye, head, and facial tissues. In this study, TG corneal neurons were retrogradely labeled and then accurately selected and pooled via FACS for RNA sequencing, which may provide more accurate neuronal biological information acquisition.

Neuron injury may be induced by varied pathogenesis, such as ERS, trauma, oxidative stress, and inflammation (Burnett and Zager, 2004; Wang et al., 2018). The cellular ER environment is sensitive to stresses which can impair folding of ER proteins and accumulation of misfolded ER proteins, resulting in trigger ERS (Belmont et al., 2010). Notably, ERS is involved in diabetic retinopathy and orofacial inflammatory pain (Yang et al., 2014; Shruthi et al., 2017) and may induce corneal epithelial damage in an *in vitro* hyperosmotic model (Wang et al., 2016). Based on the KEGG pathway reactome analysis, ERS- and apoptosis-related genes as well as proteins were found to significantly changed in the TGs of the eDED mice. Four ERS- and apoptosis-related genes with increased expression—namely, the GRP78, GRP94, XBP1, and CHOP genes—were further verified by qPCR. GRP78, GRP94 and XBP1 are highly relevant to neuronal ERS (Fu et al., 2020; Zhou et al., 2022), which is also upstream of CHOP mRNA expression in cell apoptosis (Pan et al., 2021). Therefore, ERS plays a vital role in TG corneal neuron injury in eDED. Importantly, cold, heat or mechanical receptors could be overwhelmingly activated in DED corneal nerve (Bron et al., 2017), which leads to abnormal corneal sensation. Transient receptor potential (TRP) channels can be excited by cold and heat, which are richly expressed in nerve endings (Li et al., 2019). Activation of TRP channels could lead to neuron ERS (Huang et al., 2018). Furthermore, DED corneal epithelium could release inflammatory factors (such as TNF-α, IL-1β) (Menon et al., 2021), which could activate TRP channels (Yap et al., 2020). Thus, TG corneal neuron ERS maybe induced by the stimulated nerve ending receptors triggered by irritants and corneal inflammatory factors in DED.

In this study, an ERS inhibitor was applied to eDED mice. Our results showed that ERS inhibition greatly alleviated TG corneal neuron and corneal epithelium injury. In *in vitro* hyperosmotic cell culture, TG neuronal damage was also decreased by treatment with ERS inhibitors. Consistently, ERS inhibition provides effective protection against various diseases, such as rescuing neuronal death in ischemia brain, promoting insulin sensitivity in adipose tissue, and reducing myocardial cell apoptosis in diabetic cardiomyopathy by alleviating cell damage and improving ER function (Zhang et al., 2014; Guo et al., 2017; Idari et al., 2021). In the current study, ERS displayed an important role in the TG corneal neuron injury in DED.

In summary, the current research demonstrated DED in TG corneal neuron injuries through ERS. Interventions aiming at ERS inhibition may provide a promising therapeutic approach to the treatment of DED.

## Data availability statement

The original contributions presented in the study are publicly available. This data can be found in the Genome Sequence Archive repository, accession number: CRA010234 available at https://ngdc.cncb.ac.cn/gsa/s/1N4NTL9T.

## Ethics statement

The animal study was reviewed and approved by Institutional Animal Care and Use Committee at Zhongshan Ophthalmic Center at Sun Yat-sen University.

## Author contributions

JZ contributed to investigation and original draft preparation. HL contributed to reviewing and editing and supervision. FL contributed to methodology and supervision. KW contributed to supervision and validation. SY contributed to data curation. SZ contributed to conceptualization, project administration, funding acquisition, and reviewing and editing manuscript. All authors contributed to the article and approved the submitted version.
