# Corrigendum: Involvement of endoplasmic reticulum stress in trigeminal ganglion corneal neuron injury in dry eye disease

**DOI:** 10.3389/fnmol.2024.1417118

**Published:** 2024-05-01

**Authors:** Jinyu Zhang, Hongbin Lin, Fengxian Li, Kaili Wu, Shuangjian Yang, Shiyou Zhou

**Affiliations:** ^1^State Key Laboratory of Ophthalmology, Zhongshan Ophthalmic Center, Sun Yat-sen University, Guangdong Provincial Key Laboratory of Ophthalmology and Visual Science, Guangzhou, China; ^2^Department of Anesthesiology, Zhujiang Hospital of Southern Medical University, Guangzhou, China; ^3^Guangdong Institute for Vision and Eye Research, Guangzhou, China

**Keywords:** dry eye disease, environmental dry eye disease, corneal neuron RNA-sequencing, endoplasmic reticulum stress, trigeminal ganglion corneal neuron injury

In the published article, there was an error in the abstract.

This sentence previously stated:

“Our results revealed that TG corneal neuron injury but not apoptosis in DED”

The corrected sentence appears below:

“Our results revealed that there is TG corneal neuron injury but not neuron apoptosis in DED”

The authors apologize for this error and state that this does not change the scientific conclusions of the article in any way. The original article has been updated.

