# Supplementary material for: Involvement of endoplasmic reticulum stress in trigeminal ganglion corneal neuron injury in dry eye disease
Source: Front Mol Neurosci. 2023 Mar 24;16:1083850. doi: 10.3389/fnmol.2023.1083850 (PMC10080667; doi:10.3389/fnmol.2023.1083850)
Supplement: Supplementary file 1 [file Data_Sheet_1.docx]

**Supplementary Materials**

**Methods and materials**

1. **Tear volume test**

Tear volume was measured without anesthesia with phenol red cotton threads (BWN-JM-FHMX01, Tianjin Jingming, Tianjin, China). Briefly, the threads were held with forceps and applied gently to the mice lateral canthus for 30 s (Li et al., 2019). The wetting threads were measured in millimeters under a dissection microscope.

1. **TUNEL assay**

TG frozen sections were used One Step TUNEL Apoptosis Assay Kit (C1086, Beyotime, China) according to the instruction (Shi et al., 2021). Then, sections were imaged using a confocal microscope (Nikon, AX, Japan) and analyzed using NIS-Elements software (v.5.21.00, Laboratory Imaging s.r.o., Czech Republic).

**Supplementary Figures**

**
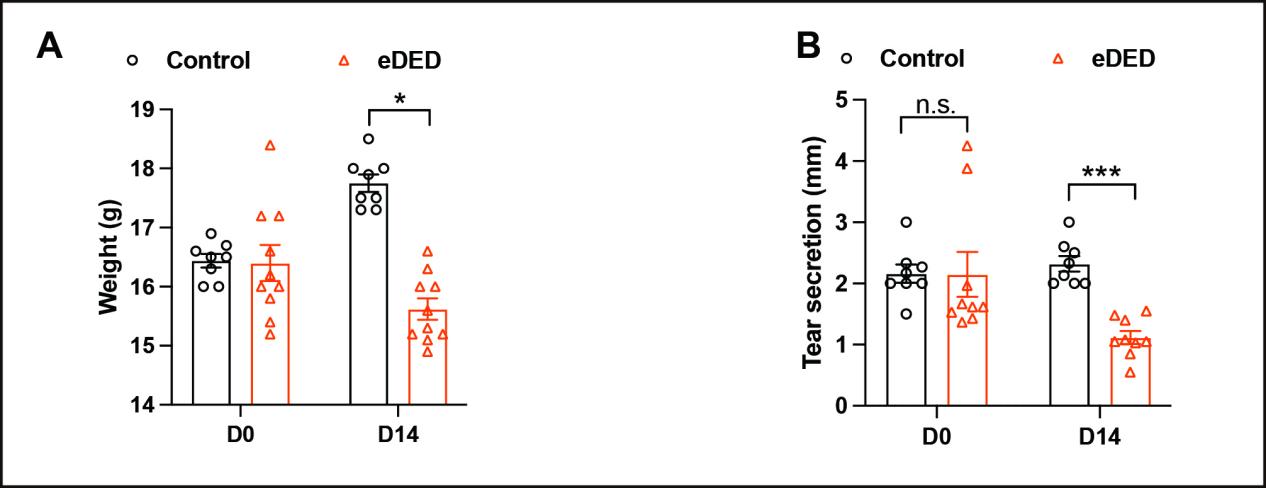
**

**Supplementary Figure 1. Weight and tear volume in eDED mice building.** (A) Bar graph showing the mice body weight in the control (n=8) and eDED (n=10) groups on day 0 and 14. (B) Bar graph showing the tear volume in the control (n=8) and eDED (n=9) groups on day 0 and 14. Data are represented as mean±SEM; **P* < 0.05, ****P* < 0.001; n.s.: not significant. eDED: environmental dry eye disease.


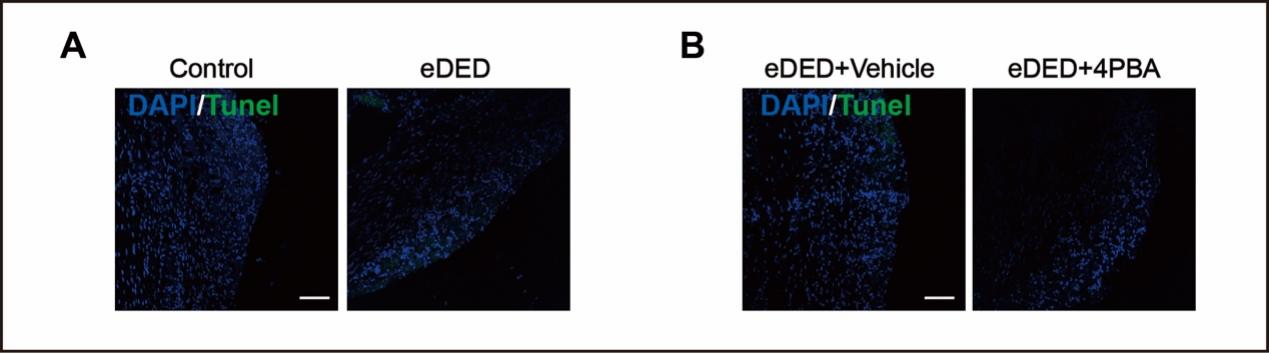


**Supplementary Figure 2. No apoptotic TG neuronal cell in eDED.** (A) Representative fluorescent images showing no apoptotic TG neuron (green) in the control and eDED groups (scale bar: 100 μm). (B) Representative fluorescent images showing no apoptotic TG neuron (green) in the eDED with and without 4-PBA treatment group; scale bar: 100 μm. eDED: environmental dry eye disease, TG: trigeminal ganglion.

**References:**

Li, F., Yang, W., Jiang, H., Guo, C., Huang, A.J.W., Hu, H., and Liu, Q. (2019). TRPV1 activity and substance P release are required for corneal cold nociception. Nat Commun 10, 5678.

Shi, Y., Xu, Y., Yao, J., Yan, C., Su, H., Zhang, X., Chen, E., and Ying, K. (2021). MTHFD2 promotes tumorigenesis and metastasis in lung adenocarcinoma by regulating AKT/GSK-3beta/beta-catenin signalling. J Cell Mol Med 25, 7013-7027.
