# Supplementary material for: Involvement of endoplasmic reticulum stress in trigeminal ganglion corneal neuron injury in dry eye disease
Source: Front Mol Neurosci. 2023 Mar 24;16:1083850. doi: 10.3389/fnmol.2023.1083850 (PMC10080667; doi:10.3389/fnmol.2023.1083850)
Supplement: Supplementary file 2 [file Table_1.DOCX]

| **Supplementary Table 1 Genes of two KEGG pathways** | |
| --- | --- |
| **Cell growth and death pathway** | **Folding, sorting and degradation pathway** |
| Dbf4 | Sec61g |
| Irf9 | Klhl9 |
| Bax | Gm11808 |
| Bid | Hspbp1 |
| Ppp1cc | Eno1 |
| Hipk1 | Bcl2 |
| Macroh2a1 | Elob |
| Cacna1d | Sae1 |
| H2M3 | Ubqln2 |
| Ppp2r5c | Yod1 |
| Ywhah | Sem1 |
| Ywhae | Dcp1b |
| Ctsb | Psmd7 |
| Gsk3b | Adrm1 |
| Adcy5 | Cdc16 |
| Hsp90ab1 | Lsm7 |
| Cdc23 | Anapc13 |
| Jak2 | Lsm3 |
| Chmp6 | Edem1 |
| Il6 | Anapc5 |
| Xiap | Ddost |
| Cd82 | Vcp |
| Nfatc2 | Nsfl1c |
| Slc7a11 | Pdia3 |
| Nras | Ube2e3 |
| Lmna | Hspa5 |
| Anapc5 | Atf6 |
| Mad1l1 | Xiap |
| Actb | Psmd13 |
| Casp2 | Dcp2 |
| Itpr1 | Cdc23 |
| Itpr2 | Cul2 |
| Tnfrsf1a | Hsp90ab1 |
| Rps6ka3 | Dad1 |
| Rbl2 | Dcp1a |
| Tradd | Spcs1 |
| Chek1 | Ssr1 |
| Tfdp2 | Pfkp |
| Tgfbr2 | Psmc1 |
| Gm28042 | Pdia6 |
| Anapc13 | Rps27a |
| Becn1 | Btg2 |
| H2az1 | Canx |
| Cdc16 | Ubb |
| Chmp4b | Psmd11 |
| Cpeb3 | Psma2 |
| Ppp1ca | Ube2g2 |
| Spata2 | Psmd4 |
| Ftl1 | Bax |
| Jun | Keap1 |
| Mdm4 | Psmc3 |
| Bcl2 |  |
| Cycs |  |
| Bub3 |  |
| Ppia |  |
| Rprm |  |
| Lin52 |  |
| Lin54 |  |

| **Supplementary Table 2 Total TGs number of a mouse after FACS** | |
| --- | --- |
| **Samples** | **Number** |
| 1 | 4.5 x10^4 |
| 2 | 5.0 x10^4 |
| 3 | 5.0 x10^4 |
| 4 | 4.5 x10^4 |
| 5 | 4.0 x10^4 |
| 6 | 4.0 x10^4 |
| Mean | 4.5 x10^4 |
| SEM | 3.8 x10^3 |

TG: trigeminal ganglion; FACS: fluorescence-activated cell sorting
